# Antimicrobial Effects and Resistant Regulation of Magnolol and Honokiol on Methicillin-Resistant *Staphylococcus aureus*

**DOI:** 10.1155/2015/283630

**Published:** 2015-08-19

**Authors:** Su Young Kim, Ju Kim, Seung-Il Jeong, Kwang Yeop Jahng, Kang-Yeol Yu

**Affiliations:** ^1^Jeonju Biomaterials Institute, Jeonju, Jeonbuk 561-360, Republic of Korea; ^2^Department of Life Science, Chonbuk National University, Jeonju, Jeonbuk 561-756, Republic of Korea

## Abstract

The antimicrobial killing activity toward methicillin-resistant *Staphylococcus aureus* (MRSA) has been a serious emerging global issue. In a continuing search for compounds with antibacterial activity against several microorganisms including *S. aureus* and MRSA, an *n*-hexane extract of *Magnolia officinalis* was found to contain magnolol. This compound exhibited potent activity against *S. aureus*, standard methicillin-susceptible *S. aureus* (MSSA), and MRSA as well as clinical MRSA isolates. When combined with oxacillin, the antibacterial activities of magnolol and honokiol against the MRSA strain were increased compared to single treatment without antibiotics at 10 *µ*g/mL and 25 *µ*g/mL, respectively. These activities of magnolol and honokiol were dose dependent. Also, magnolol showed synergistic effects with oxacillin against 13 clinical isolates of MRSA. It was determined that magnolol and honokiol had a synergistic effect with oxacillin against MRSA strain. Furthermore, the magnolol inhibited the expression of the resistant genes, *mecA, mecI, femA*, and *femB*, in mRNA. We concluded that the antibacterial activity of magnolol against MRSA strain is more related to the *mecI*'s pathway and components of the cell wall than *mecR1*. Therefore, the results obtained in this study suggest that the combination of magnolol and antibiotics could lead to the development of new combination antibiotics against MRSA infection.

## 1. Introduction

*Staphylococcus aureus*, a Gram-positive coccus, is an important human nosocomial pathogen, which causes life-threatening systemic infections such as pneumonia, osteomyelitis, endocarditis, and septicemia [1]. Today, the ongoing emergence of multidrug-resistant bacteria such as methicillin-resistant* Staphylococcus aureus* (MRSA) and the infectious diseases caused by them are serious globally [2]. Staphylococcal resistance to wide spectrum *β*-lactam antibiotics, such as methicillin, cefotaxime, and oxacillin, emerged soon after the introduction of the first drug in this class and cannot be treated with conventional *β*-lactams, making glycopeptides (vancomycin or teicoplanin) the only therapeutic solution [3, 4]. Particularly worrying is the increase in incidence of multidrug-resistant organisms such as MRSA and VRSA. In recent years, emergence of MRSA strains with reduced susceptibility to glycopeptides has emphasized the need and new approaches for novel effective drugs to combat these problems [5]. Therefore, combination therapy is often useful for patients with serious infections caused by drug-resistant pathogens [6]. In an attempt to overcome the serious problem of widespread multidrug MRSA, it is essential to study the new antibacterial agents in combination with the existing antibiotics [3]. The use of combination therapy can broaden the spectrum of antimicrobial activity, can minimize the emergence of resistant organisms, and can sometimes result in synergistic antibiotic interactions, thereby exhibiting antimicrobial activity greater than would be expected from each antimicrobial drug individually [7–9].

The herb* Magnolia officinalis *has been used for the treatment of diarrhea, acute pain, coughs, diarrhea, and urinary problems. Magnolol and honokiol are main polyphenol compounds of the bark of this medicinal plant and have a variety of pharmacological activities. Several recent reports identified and proved that magnolol and honokiol have several medicinal functions such as antioxidative, anti-inflammatory, and antitumor [10–12]. In addition, magnolol and honokiol have been known to show high antimicrobial activity against several microorganisms such as fungi* Propionibacterium *sp.* and S. aureus* [10, 13–18]. Recent research reported that magnolol inhibited the transcription of* hla* (the gene encoding *α*-toxin) in both MSSA and MRSA, resulting in a reduction of *α*-toxin production and hemolytic activities [19].

Methicillin, a *β*-lactam antibiotic, acts by inhibiting the transpeptidation domain of penicillin-binding proteins (PBPs) that are involved in the synthesis of peptidoglycan. Without cross-linking reaction of the peptidoglycan, the cell wall becomes mechanically weak, some of the cytoplasmic contents are released, and the cell dies [20]. Resistant strains typically produced an enzyme, called a *β*-lactamase, which inactivated the *β*-lactam. However,* S. aureus* can become resistant to methicillin and other *β*-lactam antibiotics through the expression of a foreign PBP, PBP2a (a low-affinity penicillin-binding protein), that is resistant to the action of methicillin but which can perform the functions of the host PBPs [20].

The mechanism of resistance to methicillin is very complex, and it is not completely characterized yet. However, it may be due to the overproduction of *β*-lactamase [20]: the expression of* mecA* producing PBP2a (also referred to as PBP2′) showed a low affinity to *β*-lactam antibiotics [21], and the change of PBP type [22, 23] detected factors other than the expression of the* mecA* gene that could control the induction of PBP2a production in* S. aureus* E 67-0 and termed it* mecR*; this* mecR* is present in some* S. aureus*, suppresses the synthesis of PBP2a present in a certain genetic background, and mediates the influence on the expression of methicillin resistance. This* mecR* consists of two genes,* mecI *(the gene encoding a transcriptional regulator (MecI)) and* mecR1 *(the gene encoding a membrane-bound signal transduction protein (MecR1)), located in the upper stream of the* mecA* gene [24]. The* mecR1 *is activated upon the contact with *β*-lactam that is equivalent to an inducing factor with MRSA and its signal binds to the promoter region of* mecA* and is transduced to* mecI* that suppresses transcription, and thus the suppression is cleared.

In addition, previous studies [20, 25] reported that the* femA *and* femB *genes play important roles in building the pentaglycine crossbridges that link the glycan chains together.

The situation is that the action mechanism of the* femA* gene is not elucidated yet; nevertheless, it is thought that it does not influence PBP2a synthesis. Significantly, inactivation of either* femA* or* femB* also results in a large reduction in methicillin resistance by the interference with the crossbridge length and the secretion of virulence factors which could limit the ability of the cell to cause infection [20]. The* femA* gene is involved in the cell wall, especially glycine content of peptidoglycan and peptidoglycan biosynthesis of* S. aureus*, and mediates the effect on drug sensitivity and thus it is involved in methicillin resistance [25, 26].

In the present study we investigated antimicrobial effects and resistant regulation of magnolol and honokiol against MRSA strains, for potential application as a natural product agent. To the best of our knowledge, this is the first work on the combined effect of magnolol and honokiol using MIC method and transcriptional regulation study of resistant gene to substantiate the synergistic antimicrobial activity of compound/antibiotics combination against MRSA and clinical MRSA isolates.

## 2. Materials and Methods

### 2.1. Reagents

Oxacillin, honokiol (≥98%) [3V,5-di-2-propenyl-(1,1V-biphenyl)-2,4V-diol], and magnolol (≥95%) [5V,5-di-2-propenyl-(1,1V-biphenyl)-2,2V-diol] were purchased from Sigma. Magnolol and honokiol were dissolved in 100% EtOH.

### 2.2. Plant Materials, Extraction, and Isolation

**Figure 1:**
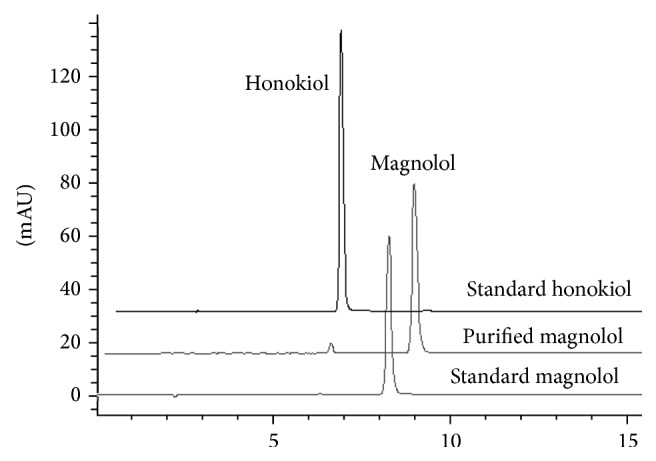
Comparison of the purified magnolol isolated from* Magnolia officinalis *with standard compounds by HPLC.

Magnolol was purified from the bark of* M. officinalis *as described previously [27]. In brief, the methanol extracts were concentrated and lyophilized with a rotary evaporator and partitioned between water and* n*-hexane. The* n*-hexane fraction was then applied to a silica gel column eluted with an* n*-hexane: acetone (8 : 11, v/v) mixture. The* n*-hexane layer was concentrated, and magnolol was directly obtained by prep-HPLC of* n*-hexane layer. The isolated magnolol was identified by thin-layer chromatograph and mixed melting point determination in comparison with authentic magnolol (Wako, Osaka, Japan). The purity (≥98%) of magnolol was determined by high-performance thin-layer chromatograph (Figure 1).

### 2.3. Bacteria

The bacteria used in this study were* Bacillus subtilis* (ATCC 6633),* Escherichia coli *(ATCC 8739)*, Propionibacterium acnes *(ATCC 6919)*, Staphylococcus aureus *(ATCC 6538), standard methicillin-susceptible* Staphylococcus aureus *(MSSA, ATCC 25923), methicillin-resistant* Staphylococcus aureus *(MRSA, ATCC 33591), and clinical MRSA isolates used in [28, 29]. All strains were cultured at 34°C for 24 h with tryptic soy medium (Difco) under aerobic conditions before the assay. After culturing all strains on Mueller-Hinton agar (Difco, Detroit, MI), the cells were resuspended in Mueller-Hinton broth (Difco) to give 10^8^ colony-forming units/mL [29]. The MRSA strains were defined on the basis of the occurrence of the* mecA* gene and of their resistance to ampicillin and methicillin, according to the guidelines of the National Committee for Clinical Laboratory Standards [29].

### 2.4. Antibacterial Activity Test and Determination of MICs

All strains were grown in tryptic soy broth for 24 hr at 34°C. The* S. aureus* suspensions were adjusted to the OD 0.3 (approximately 1 × 10^8^ CFU/mL) and treated with samples. After 24 hr incubation, 200 *μ*L of MRSA suspension was transferred to 96-well plate to measure O.D. by microplate reader (PerkinElmer, multilabel counter). The data were reported as the minimum inhibitory concentrations (MICs), the lowest concentration of magnolol and honokiol inhibiting visible growth after 24 hr of incubation 34°C [30]. The MICs of oxacillin, magnolol, and honokiol were also determined and similarly defined as the lowest concentration at which no visible bacterial growth was observed.

### 2.5. RT PCR and Real-Time PCR

MRSA (ATCC 33591) strain was grown in TSB at 34°C with the graded subinhibitory concentrations of magnolol and honokiol to the postexponential growth phase. Total RNA was isolated from the strains with Trizol reagent (Molecular Research Center, Inc.) in accordance with the manufacturer's specifications. The RNase-free DNase I (Takara) treatment was carried out to remove contaminating DNA. First-strand cDNA was synthesized using reverse transcription system (Promega) using 1 *μ*g total RNA. Ten percent of the RT product was added to a PCR reaction. 27 PCR cycles were followed by denaturation at 95°C and extension at 72°C. Primer sequences are as follows [31]: GAPDH F: 5′-TGACACTATGCAAGGTCGTTTCAC-3′, R: 5′-TCAGAACCGTCTAACTCTTGGTGG-3′;* mecA* F: 5′-GTAGAAATGACTGAACGTCCGATAA-3′, R: 5′-CCAATTCCACATTGTTTCGGTCTAA-3′;* mecI* F: 5′-CTGCAGAATGGGAAGTTATG-3′, R: 5′-ACAAGTGAATTGAAACCGCC-3′;* mecR1* F: 5′-AAGCACCGTTACTATCTGCACA-3′, R: 5′-GAGTAATTTTGGTCGAATGCC-3′;* femA* F: 5′-CATGATGGCGAGATTACAGGCC-3′, R: 5′-CGCTAAAGGTACTAACACACGG-3′;* femB* F: 5′-TTACAGAGTTAACTGTTACC-3′, R: 5′-ATACAAATCCAGCACGCTCT-3′. The real-time PCR reactions were performed in a 20 *μ*L final volume and contained SYBR master mix (Quantace), as recommended by the manufacturer. The reactions were carried out by using the Rotor-Gene 6000 (Corbett Life Science). Cycling parameters were as follows: 95°C for 10 min; 40 cycles at 95°C for 10 s, 60°C for 15 s, and 72°C for 20 s. All samples were analyzed in triplicate, and the GAPDH gene was used as an internal control housekeeping gene to normalize the levels of expression between samples. The real-time PCR data were analyzed by the (ΔΔCt) method using the Rotor-Gene comparative concentration utility.

### 2.6. Statistical Analysis of the Results

Experimental data were analyzed using SigmaPlot 10.0 statistical software. Data are expressed as the mean ± SD. Statistical differences were examined using the independent Student *t*-test. A *p* value less than 0.05 was considered statistically significant.

## 3. Results and Discussion

Infection caused by MRSA poses a serious global problem, because MRSA strains are resistant to many antibiotics in the hospital environment. Particularly worrying is the increase in the incidence of multidrug-resistant organisms such as MRSA and VRSA. Thus novel antimicrobials for new approaches to resolve these problems are urgently needed. In a continuing search for the compounds with antibacterial activity against MRSA*, Magnolia officinalis *was investigated. The MeOH extract of* M. officinalis* was fractionated into hexane, chloroform, ethyl acetate, and* n*-butanol soluble fractions. Of these, only the hexane extract showed notable antibacterial activity against the MRSA strain. Therefore, aiming to identify the active substances, the hexane fraction was submitted to column chromatography in silica gel. A needle, white compound was purified by repeated prep-HPLC as the active constituent against the bacteria. On the basis of the foregoing findings, the compound was determined to be named as magnolol. Comparative chromatograms of the purified magnolol, the standard magnolol, and honokiol showed that the purity of the purified magnolol, the standard magnolol, and honokiol was 96%, 99%, and 98%, respectively. The result also showed that the HPLC retention time of magnolol was different from honokiol (Figure 1). Also, the different HPLC retention time of magnolol from honokiol, respectively, showed that they are different compounds.

**Figure 2:**
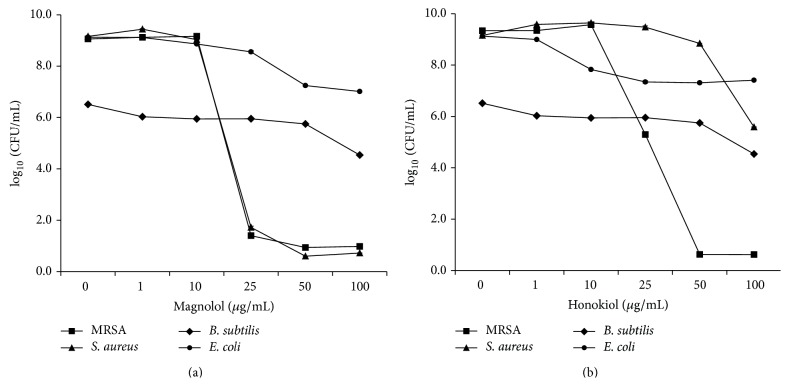
Antimicrobial activities of magnolol (a) and honokiol (b) against* Staphylococcus aureus* (ATCC 6538), methicillin-resistant* Staphylococcus aureus *(MRSA, ATCC 33591),* Bacillus subtilis* (ATCC 6633), and* Escherichia coli *(ATCC 8739). All strains were cultured at 34°C for 24 h with tryptic soy medium (Difco) under aerobic conditions before the assay. For antibacterial activity test, Mueller-Hinton medium (Difco, Detroit, MI) was used.

In a continuing search for the compounds with antibacterial activity against several microorganisms including* S. aureus,* MRSA (ATCC 33591),* B. subtilis, *and* E. coli, *an* n*-hexane extract of* M. officinalis* showed antimicrobial activities. Magnolol showed antibacterial activities against* S. aureus*, MRSA, and 13 clinical MRSA isolates (Figure 2(a) and Table 1). According to previous studies [13, 14], magnolol and honokiol showed antibacterial activities against* P. acnes *(data not shown) and* B. subtilis *except* E. coli *(Figure 2). Moreover, the growth of MRSA cultured with magnolol was significantly decreased in the range of 10–25 *μ*g/mL similar to* S. aureus. *As shown in Figure 2(b), the growth of MRSA cultured with honokiol was significantly decreased in the range of 10–50 *μ*g/mL. However, there was no obvious antimicrobial effect of honokiol against* S. aureus, B. subtilis, *and* E. coli *at 50 *μ*g/mL. Magnolol showed better antibacterial activity against* S. aureus *and MRSA than honokiol. These data suggest that magnolol may be useful to treat* S. aureus *and MRSA infections.

**Figure 3:**
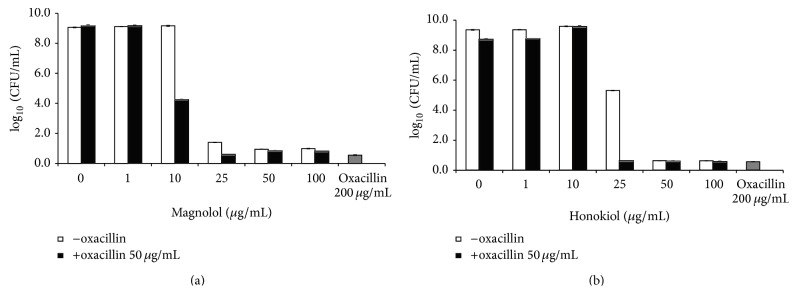
Synergistic effect of magnolol and honokiol with oxacillin against MRSA. Oxacillin 50 *μ*g/mL was used for synergistic effect. MRSA strain was grown in tryptic soy broth for 24 hr at 34°C.

The antibacterial activities of magnolol and honokiol with antibiotics against MRSA were shown in Figure 3. When combined with 50 *μ*g/mL oxacillin, the antibacterial activities of magnolol and honokiol against the MRSA standard strain were increased compared to single treatment without antibiotics at 10 *μ*g/mL and 25 *μ*g/mL, respectively. These activities of magnolol and honokiol were dose dependent. Also, magnolol exhibited potent activities against the standard MSSA and MRSA as well as the clinical isolates of MRSA. As shown in Table 1, magnolol showed more significant antibacterial activity with oxacillin than single treatment without antibiotics against 13 clinical isolates of MRSA at 10 *μ*g/mL. These results determined that magnolol and honokiol had a synergistic effect with oxacillin against MRSA strain. These data suggest that magnolol and honokiol are potentially useful for the treatment of drug-resistant* S. aureus *infections when used in combination with lower concentration of antibiotics. Against the MRSA standard strain, the MICs of magnolol and oxacillin were 20 *μ*g/mL and 177 *μ*g/mL, respectively (Table 2). The MIC of magnolol, in combination with 50 *μ*g/mL oxacillin, was 16 *μ*g/mL against the MRSA standard strain. Also, MIC of honokiol was 33 *μ*g/mL, and the MIC of honokiol, in combination with 50 *μ*g/mL oxacillin, was 23 *μ*g/mL against the MRSA standard strain. There was no significant synergistic effect between single treatment of magnolol and the combination treatment with antibiotics when compared to MICs.

In the present study, five antibiotics were chosen according to their different mechanism of action against MRSA. Both oxacillin and ampicillin from penicillin class and cefoxitin from cephalosporin class are the inhibitors of cell wall biosynthesis, while chloramphenicol and tetracycline are protein inhibitors [3, 32]. The combination of antimicrobial agent acts on the different target site of bacteria that could lead to synergistic effect [33]. Antibacterial activities of magnolol and honokiol with the combination of five representative antibiotics, oxacillin (Ox), ampicillin (Amp), chloramphenicol [33], tetracycline (Tet), and cefoxitin (Cef), were evaluated against standard MRSA strain (Table 2). When combined with 50 *μ*g/mL oxacillin, the magnolol showed the best synergistic effect (77%) compared to other antibiotics against MRSA strain. On the other hand, when combined with 25 *μ*g/mL chloramphenicol and 10 *μ*g/mL cefoxitin, the honokiol showed the best synergistic effect (93%) compared to other antibiotics. It was shown from these results that magnolol and honokiol had a synergistic effect with several representative antibiotics as well as oxacillin against MRSA strain. These data suggest that magnolol and honokiol are potentially useful for the treatment of multidrug-resistant* S. aureus *infections when used in combination with lower concentration of antibiotics.

**Figure 4:**
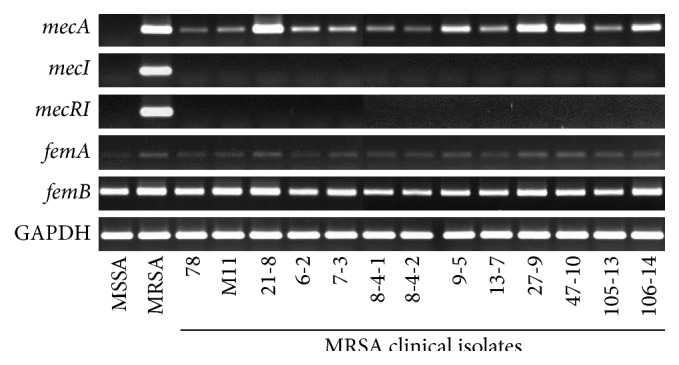
RT-PCR analysis of the level of* mecA*,* mecI*,* mecR1*,* femA,* and* femB* mRNA in* S. aureus* strains. All strains were grown in tryptic soy broth for 24 hr at 34°C.

To investigate the resistant regulation of magnolol and honokiol against MRSA strains, we tried the RT-PCR (reverse transcriptase-polymerase chain reaction) analysis of resistant genes involved in MRSA resistance to *β*-lactam antibiotics.  Figure 4 shows the results of resistant gene expression for MRSA and clinical MRSA isolates using RT-PCR. The standard MRSA (ATCC 33591) was* mecA*-positive and the standard MSSA (ATCC 25923) was* mecA*-negative. We could not confirm the gene expression of* mecA, mecI,* and* mecR1* in* S. aureus* (ATCC 6538) as well as MSSA (ATCC 25923), but we could see these genes appear clearly in MRSA (ATCC 33591). The* mecA* gene expressed all MRSA strains including standard MRSA (ATCC 33591) and 13 clinical MRSA isolates. For further study about the resistant gene regulation of magnolol and honokiol, we selected the standard MRSA (ATCC 33591) expressing all resistant genes.

**Figure 5:**
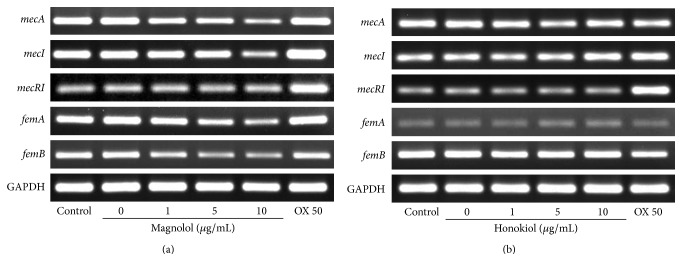
RT-PCR analysis of the level of* mecA*,* mecI*,* mecR1*,* femA,* and* femB* mRNA in MRSA (ATCC 33591) strain treated with the indicated concentrations of the magnolol (a) and honokiol (b). GAPDH was employed as an internal control and oxacillin 50 *μ*g/mL (OX 50) was used in this study. MRSA strain was grown in tryptic soy broth for 24 hr at 34°C.

**Figure 6:**
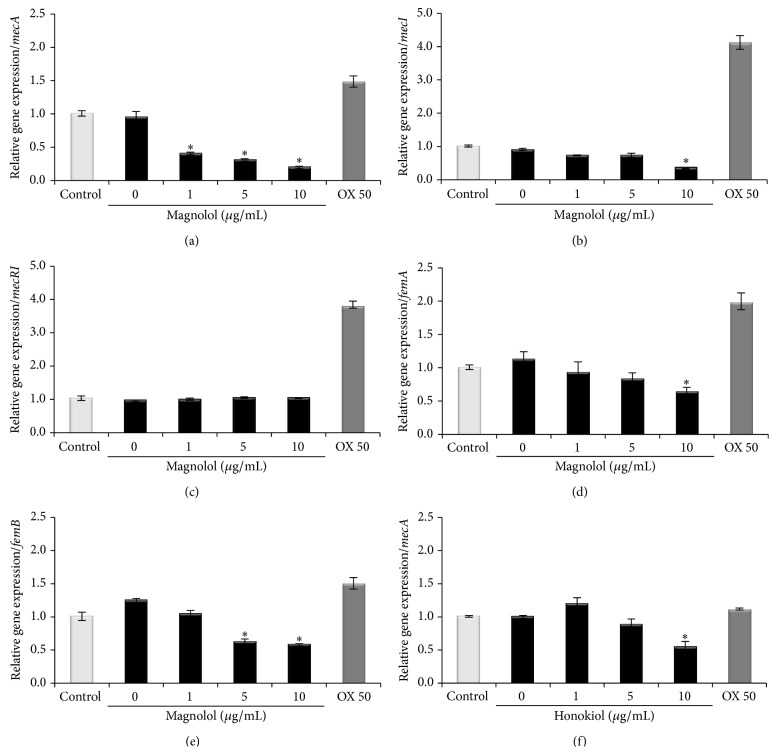
Relative gene expression of* mecA*,* mecI*,* mecR1*,* femA,* and* femB* gene in MRSA (ATCC 33591) strain treated with the indicated concentrations of the magnolol ((a)–(e)) and honokiol (f). Oxacillin 50 *μ*g/mL (OX 50) was used in this study. MRSA strain was grown in tryptic soy broth for 24 hr at 34°C. Values represent the mean and standard error of three independent experiments. Statistical differences were examined using the independent Student *t*-test. A *p* value less than 0.05 was considered statistically significant. *∗* represents *p* < 0.05.

As shown in Figure 5, the expression of the* mecA* gene was inhibited in a dose-dependent manner by the magnolol and was weekly decreased by honokiol. Also,* femA *and* femB *genes known to be related with the composition of cell wall also exhibited that magnolol inhibited gene expression. Real-time PCR was carried out to assess the transcriptional level of the resistant genes,* mecA, mecI, mecR1, femA,* and* femB, *after treatment with subinhibitory concentrations of magnolol and honokiol. As shown in Figure 6, magnolol significantly inhibited the transcription of* mecA, mecI, femA,* and* femB *in MRSA (ATCC 33591). When cultured with 1 *μ*g/mL, 5 *μ*g/mL, and 10 *μ*g/mL of magnolol, the transcriptional levels of* mecA *in MRSA strain were decreased by 0.40-, 0.30-, and 0.19-fold, respectively. Also, the transcriptional levels of* mecI *were decreased at 5 *μ*g/mL and 10 *μ*g/mL by 0.70- and 0.32-fold, respectively. The transcriptional levels of* femA *and* femB *were decreased at 10 *μ*g/mL magnolol by 0.63- and 0.59-fold, respectively. When cultured with 10 *μ*g/mL of honokiol, the transcriptional level of* mecA *in MRSA strain was decreased by 0.54-fold. The* mecA *gene was affected in a dose-dependent manner by magnolol and honokiol at the transcriptional level in a dose-dependent manner. This was due to the regulation of methicillin resistance by magnolol and honokiol thereby inhibiting* mecA* gene expression that could control the induction of PBP2a production. However,* mecR1* gene did not show the effectiveness against MRSA strain, so we concluded that the antibacterial activity of magnolol against MRSA strain is more related to* mecI*'s pathway than* mecR1*.

Magnolol and honokiol, main compounds isolated from the bark of* M. officinalis*, increase the susceptibility of MRSA isolates to *β*-lactams either by affecting the expression of the* mecA* gene or by interfering with either the signalling domain of MecI or some other protein involved in signal transduction and components of the cell wall. By targeting and inhibiting the proteins, such as PBP2a, FemA, and FemB, involving the formation of these substrates, methicillin resistance can be modulated. Compounds such as magnolol and honokiol are attractive compounds to develop into therapeutic agents since these compounds already exist and are known to modulate methicillin resistance.

## 4. Conclusion

The present study investigated the antimicrobial effects and the resistant regulation mechanism of magnolol and honokiol against MRSA strains, for potential application as a natural product agent. These compounds exhibited potent activity against MSSA as well as clinical isolates of MRSA. Furthermore, magnolol and honokiol showed the synergistic effect with oxacillin against MRSA strain and reduced expression of the resistant genes,* mecA, mecI, femA,* and* femB,* in mRNA. From the study it was concluded that magnolol has the potential to restore the effectiveness of *β*-lactam antibiotics against MRSA and other strains of *β*-lactam-resistant* S. aureus*. In a view of its limited toxicity, magnolol offers the potential for the development of a valuable adjunct to *β*-lactam treatments against otherwise resistant strains of microorganisms. Therefore, the results obtained in this study suggest that the combination of magnolol and antibiotics could lead to the development of new combination antibiotics against MRSA infection.

## Figures and Tables

**Table 1:** Antibacterial activity tests of magnolol against methicillin-resistant *S. aureus* (MRSA).

*S. aureus* strain	Class	Magnolol (*μ*g/mL)^*∗*^					Oxacillin 25 *μ*g/mL + magnolol (*μ*g/mL)^*∗*^				
		0	1	10	25	50	0	1	10	25	50
ATCC25923	MSSA	+++	+++	+++	+/−	—	—	—	—	—	—
ATCC33591	MRSA	+++	+++	+++	+/−	—	+++	+++	+++	—	—
Clinical isolates											
*S. aureus* 78	MRSA	+++	+++	+++	—	—	+++	+++	—	—	—
*S. aureus* M11	MRSA	+++	+++	+++	+/−	—	+++	+++	—	—	—
*S. aureus* 21-8	MRSA	+++	+++	+++	—	—	+++	+++	+/−	—	—
*S. aureus* 6-2	MRSA	+++	+++	+++	—	—	+++	+++	—	—	—
*S. aureus* 7-3	MRSA	+++	+++	+++	+/−	—	+++	+++	—	—	—
*S. aureus* 8-4-1	MRSA	+++	+++	+++	+/−	—	+++	+++	—	—	—
*S. aureus* 8-4-2	MRSA	+++	+++	+++	+/−	—	+++	+++	—	—	—
*S. aureus* 9-5	MRSA	+++	+++	+++	+/−	—	+++	+++	—	—	—
*S. aureus* 13-7	MRSA	+++	+++	+++	—	—	+++	+++	+/−	—	—
*S. aureus* 27-9	MRSA	+++	+++	+++	—	—	+++	+++	—	—	—
*S. aureus* 47-10	MRSA	+++	+++	+++	—	—	+/−	+/−	—	—	—
*S. aureus* 105-13	MRSA	+++	+++	+++	+/−	—	+++	+++	—	—	—
*S. aureus* 106-14	MRSA	+++	+++	+++	+/−	—	+++	+++	+/−	—	—

^*∗*^+++: growing very well; +/−: growing slightly; —: no growth.

**Table 2:** The MICs and synergistic effects of the magnolol and honokiol against standard MRSA strain.

Antibacterial agent	MIC (*µ*g/mL)		Antibiotics^*∗*^				
	− Oxacillin	+ Oxacillin 50 *µ*g/mL	Ox	Amp	Cam	Tet	Cef
Oxacillin	177						
Magnolol	20	16	syn (77%)	—	syn (15%)	syn (22%)	syn (54%)
Honokiol	33	23	syn (38%)	—	syn (93%)	—	syn (93%)

^*∗*^Ox: oxacillin 50 *µ*g/mL; Amp: ampicillin 100 *µ*g/mL; Cam: chloramphenicol 25 *µ*g/mL; Tet: tetracycline 15 *µ*g/mL; Cef: cefoxitin 10 *µ*g/mL.
